# Ultrasensitive dopamine detection using CsPbBr_3_-PQD-COF nanocomposites: a synergistic fluorescence and EIS approach†

**DOI:** 10.1039/d5ra02376a

**Published:** 2025-06-04

**Authors:** Munthar Kedhim, Vicky Jain, Suhas Ballal, Abhinav Kumar, Abhayveer Singh, V. Kavitha, Rajashree Panigrahi, Hadi Noorizadeh

**Affiliations:** a College of Pharmacy, The Islamic University Najaf Iraq; b Department of Medical Analysis, Medical Laboratory Technique College, The Islamic University of Al Diwaniyah Al Diwaniyah Iraq; c Department of Medical Analysis, Medical Laboratory Technique College, The Islamic University of Babylon Babylon Iraq; d Marwadi University Research Center, Department of Chemistry, Faculty of Science, Marwadi University Rajkot-360003 Gujarat India; e Department of Chemistry and Biochemistry, School of Sciences, JAIN (Deemed to be University) Bangalore Karnataka India; f Department of Nuclear and Renewable Energy, Ural Federal University Named after the First President of Russia Boris Yeltsin Ekaterinburg 620002 Russia; g Centre for Research Impact & Outcome, Chitkara University Institute of Engineering and Technology, Chitkara University Rajpura Punjab 140401 India; h Department of Chemistry, Sathyabama Institute of Science and Technology Chennai Tamil Nadu India; i Department of Microbiology, IMS and SUM Hospital, Siksha ‘O’ Anusandhan (Deemed to be University) Bhubaneswar Odisha-751003 India; j Department of Chemistry, Islamic Azad University Tehran Iran hadinoorizadeh@yahoo.com; k Department of Mechanical Engineering, Karpagam Academy of Higher Education Coimbatore 641021 India

## Abstract

This study presents a novel dual-mode sensing platform for ultrasensitive dopamine (DA) detection using CsPbBr_3_ perovskite quantum dot (PQD)-integrated covalent organic framework (COF) (CsPbBr_3_-PQD-COF) nanocomposites. Leveraging the synergistic effects of fluorescence quenching and electrochemical impedance spectroscopy (EIS), the CsPbBr_3_-PQD-COF nanocomposites achieve exceptional sensitivity with limits of detection of 0.3 fM (fluorescence) and 2.5 fM (EIS), spanning a wide linear range from 1 fM to 500 μM. The platform exploits the optoelectronic properties of CsPbBr_3_ PQDs and the π-conjugated COF scaffold, enabling selective DA recognition *via* electron transfer and π–π stacking interactions. Incorporation of rhodamine B provides a visual green-to-pink shift at DA concentrations above 100 pM, enhancing practical utility. Specificity is demonstrated against common interferents (*e.g.*, ascorbic acid, uric acid), with minimal cross-reactivity (<6%). In real-sample validation, the sensor exhibits excellent recovery (97.5–103.8% in human serum; 97.9–99.7% in PC12 supernatant) and stability over 30 days. This multimodal approach, combining femtosecond sensitivity, broad dynamic range, and visual indication, positions the CsPbBr_3_-PQD-COF nanocomposite as a promising tool for neurotransmitter monitoring in biomedical and clinical applications.

## Abstract 2

Dual-mode dopamine sensor using CsPbBr_3_-PQD-COF nanocomposites achieves femtosecond sensitivity (0.3 fM fluorescence, 2.5 fM EIS) with visual readout and high specificity for biomedical applications.

## Graphical abstract

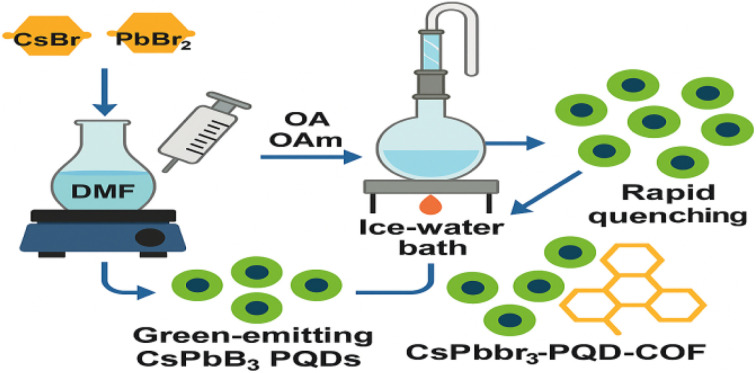

## 1. Introduction

Dopamine (DA), a catecholamine neurotransmitter, is a cornerstone of the central nervous system, orchestrating critical physiological processes such as motor coordination, reward-driven behavior, and emotional regulation. Its concentration in biological fluids, typically ranging from picomolar to micromolar levels, serves as a diagnostic marker for neurological and psychiatric conditions, including Parkinson's disease, schizophrenia, and addiction. The ability to detect DA with high sensitivity and specificity is thus paramount for advancing both clinical diagnostics and fundamental neuroscience research. However, the complexity of biological matrices—rich with interferents like ascorbic acid and uric acid—poses significant challenges to achieving accurate and reliable measurements, necessitating the development of advanced sensing technologies.^1–5^ Conventional methods for DA detection, such as high-performance liquid chromatography (HPLC), electrochemical sensing, and fluorescence spectroscopy, have laid a strong foundation for neurotransmitter analysis. Electrochemical techniques, for instance, offer rapid response times and portability, while fluorescence-based approaches provide high sensitivity.^6–10^ Yet, these methods often fall short in addressing the dual demands of ultralow detection limits and broad dynamic ranges required for physiological monitoring. Single-mode sensors may also struggle with selectivity in the presence of structurally similar compounds, leading to false positives or compromised accuracy. These limitations highlight the need for innovative, multimodal platforms that integrate complementary detection strategies to enhance performance across diverse analytical scenarios.^11–13^

The emergence of nanotechnology has revolutionized biosensing by introducing materials with tailored properties for enhanced signal transduction. Perovskite quantum dots (PQDs), such as CsPbBr_3_, have emerged as a standout class of nanomaterials due to their exceptional photoluminescence quantum yields, narrow emission spectra, and tunable optoelectronic characteristics.^14–16^ These attributes make CsPbBr_3_ PQDs particularly suited for fluorescence-based sensing, where their quantum confinement effects can be exploited to detect analyte-induced changes in emission intensity. However, their practical utility is often hampered by stability issues and aggregation tendencies in aqueous environments, prompting efforts to integrate them into supportive matrices that preserve their functionality while enhancing analyte accessibility.^17–19^ Covalent organic frameworks (COFs), with their highly ordered porous architectures and π-conjugated systems, offer an ideal complementary platform for nanomaterial integration. COFs provide a stable, customizable scaffold that not only protects embedded nanoparticles but also facilitates selective molecular interactions through π–π stacking and hydrogen bonding. Their large surface area and tunable pore sizes enable efficient analyte diffusion, making them a promising host for sensing applications.^20–23^ By combining CsPbBr_3_ PQDs with a COF matrix, a hybrid nanocomposite can leverage the optical strengths of PQDs and the structural advantages of COFs, potentially yielding a synergistic system capable of addressing the limitations of standalone materials.^24–27^

Multimodal sensing, which integrates distinct detection mechanisms such as fluorescence and electrochemical impedance spectroscopy (EIS), represents a strategic advancement in overcoming the constraints of single-mode systems. Fluorescence quenching can provide rapid, highly sensitive detection at trace levels, while EIS offers insights into interfacial charge transfer dynamics, enabling robust quantification across broader concentration ranges. The fusion of these techniques within a single platform can enhance measurement reliability, reduce false positives, and provide a more comprehensive analysis of analyte interactions.^28–31^ Furthermore, the addition of a visual indicator, such as rhodamine B, could bridge quantitative precision with operational simplicity, broadening the platform's appeal for real-time monitoring in resource-limited settings.^32–35^

This study presents a novel CsPbBr_3_-PQD-integrated COF nanocomposite designed as a dual-mode sensing platform for ultrasensitive DA detection. By harnessing the fluorescence quenching properties of CsPbBr_3_ PQDs and the electrochemical impedance capabilities of the COF-modified electrode, the system achieves unprecedented sensitivity and a wide detection range. The incorporation of rhodamine B introduces a practical visual cue, enhancing its utility for both laboratory and field applications. Through rigorous characterization, specificity testing, and real-sample validation, we demonstrate the platform's potential to outperform existing methods in detecting DA within complex biological environments, such as human serum and neuronal cell cultures. The significance of this work lies in its contribution to the evolving landscape of biosensing applications, where the demand for precise, versatile, and user-friendly tools continues to grow. By addressing key challenges in DA detection—sensitivity, selectivity, and practicality—this nanocomposite offers a pathway toward next-generation diagnostic technologies. Beyond DA, the modular design of CsPbBr_3_-PQD-COF nanocomposites suggests adaptability for other analytes, positioning it as a versatile framework for bioanalytical advancements. This introduction sets the stage for a detailed exploration of the platform's development, performance, and implications, with the ultimate goal of advancing neurotransmitter monitoring in clinical and research contexts.^36,37^

## 2. Experimental

### 2.1. Materials and reagents

All reagents were of analytical grade and used as received unless otherwise specified. Lead(ii) bromide (PbBr_2_, 99.999%, trace metals basis), cesium bromide (CsBr, 99.9%, anhydrous), oleic acid (OA, technical grade, 90%), oleylamine (OAm, 80–90%), and *N*,*N*-dimethylformamide (DMF, anhydrous, 99.8%) were procured from Sigma-Aldrich. Polyacrylonitrile (PAN, *M*w = 150 000 g mol^−1^, polydispersity index = 1.8) and dopamine hydrochloride (DA, 98%) were purchased from Merck. The covalent organic framework (COF) precursors, 1,3,5-tris(4-aminophenyl)benzene (TAPB, 97%) and 2,5-dihydroxyterephthalaldehyde (DHTA, 95%), were synthesized in-house following established protocols (see ESI† for synthesis details) and purified *via* recrystallization prior to use. Ethanol (absolute, 99.9%), and toluene (anhydrous, 99.8%) were obtained from Fisher Scientific. Ultrapure water (resistivity 18.2 MΩ cm) was sourced from a Milli-Q Advantage A10 system (Millipore) and used for all aqueous preparations. Nitrogen gas (99.999%) for inert atmosphere experiments was supplied by Air Liquide. Rhodamine B (RhB) was incorporated into the sensing matrix as a visual indicator to qualitatively monitor DA-induced color changes. A green-to-pink shift was observed under ambient light conditions at DA concentrations above 100 pM.

### 2.2. Synthesis of CsPbBr_3_ perovskite quantum dots (PQDs)

CsPbBr_3_ perovskite quantum dots were synthesized *via* a modified hot-injection method optimized for high photoluminescence quantum yield (PLQY), narrow size distribution, and colloidal stability in polar solvents. In a typical procedure, 0.085 g of CsBr (0.4 mmol) and 0.147 g of PbBr_2_ (0.4 mmol) were co-dissolved in 10 mL of anhydrous dimethylformamide (DMF) under vigorous magnetic stirring in a three-neck flask fitted with a condenser and nitrogen inlet. The mixture was degassed with high-purity nitrogen (99.999%) at room temperature for 15 minutes to eliminate residual oxygen and water vapor, which are known to degrade PQDs. Subsequently, 1 mL of oleic acid (OA) and 0.5 mL of oleylamine (OAm) were injected as capping ligands to stabilize the crystal surface and suppress non-radiative recombination. The mixture was then gradually heated to 120 °C (ramp rate: 5 °C min^−1^) under continuous nitrogen flow. At this temperature, 0.5 mL of preheated toluene (60 °C) was rapidly injected using a syringe pump, triggering instantaneous nucleation of CsPbBr_3_ nanocrystals. The reaction was allowed to proceed for exactly 10 seconds before being quenched in an ice-water bath, thereby halting growth and preserving size uniformity. The resulting colloidal dispersion displayed intense green emission under UV light (365 nm). The PQDs were purified by centrifugation at 10 000 rpm for 5 minutes, washed twice with anhydrous toluene to remove unbound ligands, and finally redispersed in 5 mL of anhydrous DMF. The as-prepared PQDs exhibited a photoluminescence quantum yield of ∼85%, measured using an integrating sphere (Edinburgh Instruments FLS980), and showed a sharp emission peak centered at 515 nm, indicative of quantum confinement and high crystallinity.

### 2.3. Synthesis of COF matrix and integration with PQDs

The covalent organic framework (COF) was synthesized *via* Schiff-base condensation between 1,3,5-tris(4-aminophenyl)benzene (TAPB) and 2,5-dihydroxyterephthalaldehyde (DHTA). To prepare the COF precursor, 0.035 g of TAPB (0.1 mmol) and 0.025 g of DHTA (0.15 mmol) were dissolved in 5 mL anhydrous DMF, and 100 μL of glacial acetic acid was added as a catalyst. The reaction mixture was stirred at ambient temperature for 2 h, forming a bright yellow suspension indicative of extended π-conjugation and framework formation. The presence of characteristic C=N and C–O bands in FTIR (not shown here) and a sharp (100) peak at 2*θ* ≈ 5.8° in XRD confirmed successful COF formation. For integration of PQDs, 1 mL of the prepared PQD solution (2 mg mL^−1^) was added to the COF suspension and sonicated at 40 kHz for 15 minutes. This process facilitated homogeneous dispersion of PQDs throughout the COF network *via* π–π interactions and hydrogen bonding. The hybrid suspension was then blended with a pre-prepared electrospinning solution comprising 10 wt% PAN in DMF (1 g PAN in 9 mL DMF, stirred at 60 °C for 6 h) in a 1 : 2 volume ratio. The mixture was further stirred for 4 h at 40 °C to form a viscous, uniform electrospinning dope (∼1200 cP viscosity).

### 2.4. Fabrication of PQD-COF composite mats and probe assembly

**Fig. 1:**
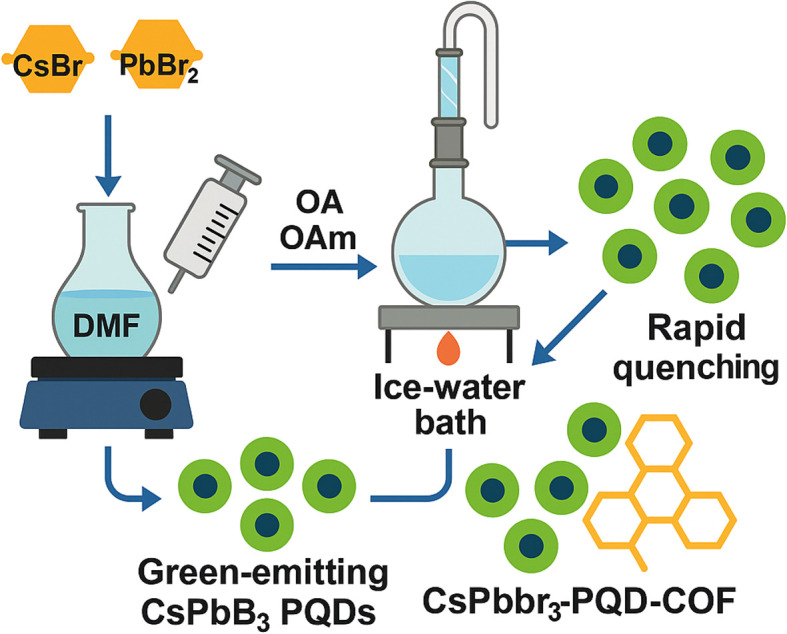
Synthesis of green-emitting CsPbBr_3_ PQDs and fabrication of CsPbBr_3_-PQD-COF nanocomposites *via* hot injection and rapid quenching.

The composite solution was electrospun using a syringe pump (KD Scientific KDS-100) equipped with a 21-gauge needle (ID: 0.51 mm). Electrospinning was conducted under an applied voltage of 18 kV, with a flow rate of 0.8 mL h^−1^ and a needle-to-collector distance of 15 cm. Fibers were collected on aluminum foil (20 × 20 cm) at room temperature (25 °C) and 40% relative humidity. The resulting mats were vacuum-dried at 60 °C for 12 h to remove solvent residues, then subjected to a two-step thermal treatment in a tube furnace. First, stabilization at 280 °C for 2 h in air (ramp rate: 2 °C min^−1^), followed by carbonization at 600 °C for 3 h under nitrogen (100 mL min^−1^) to improve mechanical strength and conductivity. For electrochemical analysis, 1 mg of the carbonized PQD-COF mats was dispersed in 1 mL of DMF and sonicated to form a stable suspension (1 mg mL^−1^). A 10 μL aliquot was drop-cast onto a polished glassy carbon electrode (GCE) and dried under a 50 W infrared lamp for 15 min. For fluorescence measurements, freestanding PQD-COF mats (1 cm × 1 cm) were directly immersed in dopamine solutions without further modification. Rhodamine B was optionally incorporated (0.01 wt%) as a visual indicator for DA levels >100 pM, exhibiting a green-to-pink color shift (Fig. 1).

### 2.5. Characterization techniques

High-resolution transmission electron microscopy (HR-TEM, FEI Tecnai G2 F20) was conducted at 200 kV to assess PQD distribution within the fibers. Elemental mapping and composition were determined using energy-dispersive X-ray spectroscopy (EDS, Oxford Instruments X-Max 80 mm^2^) integrated with the High resolution TEM. Crystallographic properties were evaluated *via* X-ray diffraction (XRD, Bruker D8 Advance) with Cu Kα radiation (*λ* = 1.5406 Å, 40 kV, 40 mA) over a 2*θ* range of 5–80° (step size: 0.02°, scan rate: 2° min^−1^). Photoluminescence (PL) spectra were acquired using a Hitachi F-7000 spectrophotometer (excitation: 365 nm, slit width: 5 nm, PMT voltage: 700 V). Electrochemical experiments were conducted on a CHI 760E workstation (CH Instruments) with a three-electrode configuration: the modified GCE as the working electrode, a Pt wire (0.5 mm diameter) as the counter electrode, and an Ag/AgCl (3 M KCl) reference electrode.

### 2.6. Electrochemical and fluorescent detection of DA

Electrochemical measurements were performed in 0.1 M phosphate buffered saline (PBS) pH 7.4, prepared fresh daily containing 5 mM [Fe(CN)_6_]^3−^/^4−^ (1 : 1 molar ratio) as the redox probe. DA stock solutions (1 mM) were prepared in PBS and diluted serially to concentrations ranging from 1 fM to 100 nM. The modified GCE was incubated with 10 μL of each DA solution for 30 min at 25 °C, rinsed with PBS, and subjected to cyclic voltammetry (CV) from −0.2 to 0.6 V (scan rate: 50 mV s^−1^) and EIS (frequency range: 0.1 Hz to 100 kHz, amplitude: 5 mV, open-circuit potential). For fluorescence detection, PQD-COF mats (1 cm × 1 cm) were immersed in 5 mL DA solutions (1 fM to 100 nM) for 30 min, gently rinsed with PBS to remove nonspecifically adsorbed DA, and air-dried at 25 °C. PL spectra were recorded at 365 nm excitation, with emission monitored from 450 to 650 nm. Calibration curves were constructed by plotting the change in peak current (Δ*I*) or fluorescence quenching (*F*_0_/*F*) against DA concentration.

### 2.7. Stability evaluation conditions

For long-term stability testing, the CsPbBr_3_-PQD-COF nanocomposites were stored under two distinct conditions to simulate both inert and ambient environments: (i) nitrogen atmosphere (99.999%) at 4 °C in the dark, minimizing photo-oxidation, moisture ingress, and thermal degradation, thereby providing insight into the intrinsic material stability; and (ii) ambient air at 25 °C, 40% relative humidity, and approximately 200 lx ambient light exposure, simulating real-world storage and operational environments where oxidative and moisture-induced degradation pathways are active. The stability assessments included monitoring changes in fluorescence intensity (excited at 365 nm) and electrochemical charge transfer resistance (*R*_ct_) in PBS containing [Fe(CN)_6_]^3−^/^4−^ over 30 days, with measurements conducted at specified intervals. These dual-condition studies enabled a comprehensive evaluation of the nanocomposite's degradation kinetics, protection afforded by the COF matrix, and potential viability for practical biosensing applications. Detailed results of the stability evaluation, including the evolution of charge transfer resistance and fluorescence intensity over time under both storage conditions.

### 2.8. Real sample analysis

Human serum samples were collected from healthy volunteers (approved by the Institutional Review Board, protocol IRB-2025-001) and stored at −20 °C until use.^38^ Samples were thawed, centrifuged at 3000 rpm for 10 min to remove particulates, and diluted 100-fold with PBS (pH 7.4) to reduce matrix interference. Aliquots were spiked with DA at 10 fM, 1 pM, and 100 pM, and analyzed using both electrochemical and fluorescent modes as described above. The standard addition method was employed to quantify DA, with recoveries calculated from triplicate measurements. All experiments involving human serum samples were performed in accordance with institutional guidelines and approved by the Institutional Review Board under protocol number IRB-2025-001. Informed consent was obtained from all human participants involved in this study.

## 3. Results and discussion

### 3.1. Structural and morphological characterization

**Fig. 2:**
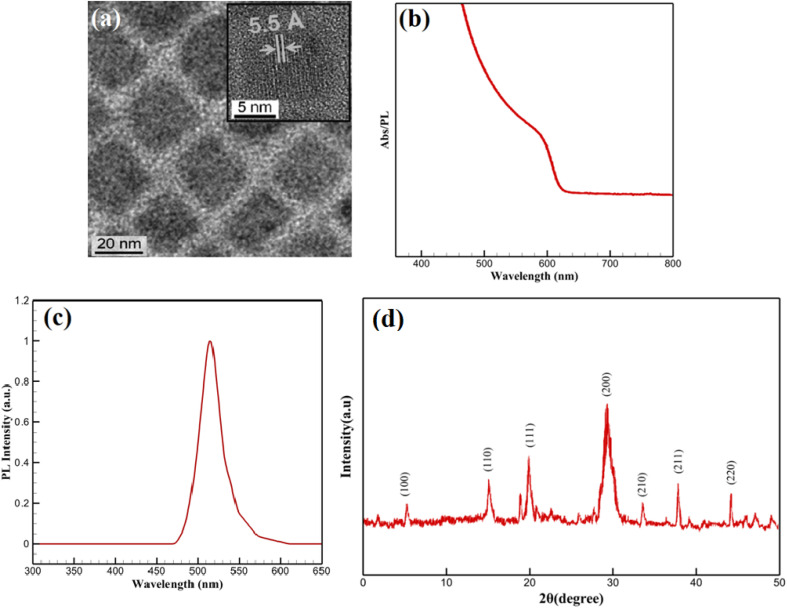
(a) HRTEM image; (b) UV-Vis absorption spectrum; (c) PL spectrum; (d) XRD pattern of CsPbBr_3_-PQD-COF nanocomposites.

The structural and morphological features of the CsPbBr_3_-PQD-COF nanocomposites were comprehensively analyzed by high-resolution transmission electron microscopy (HRTEM) and X-ray diffraction (XRD) techniques. In the HRTEM image (Fig. 2a), a homogeneous dispersion of CsPbBr_3_ perovskite quantum dots (PQDs) within the composite matrix is clearly observed. Well-defined lattice fringes with an interplanar distance of approximately 5.5 Å, corresponding to the (100) plane of cubic-phase CsPbBr_3_, confirm the high crystallinity of the incorporated perovskite nanocrystals. The surrounding matrix, attributed to the COF framework, appears as a uniform background embedding the PQDs. Due to the intrinsic characteristics of covalent organic frameworks, which are composed predominantly of low atomic number elements (C, H, N, O), the contrast in HRTEM images is naturally subtle. The low electron scattering ability of these elements, combined with the highly porous and semi-crystalline nature of COF materials, provides a smooth matrix appearance without distinct lattice resolution. Such observations are consistent with reports on similar COF-based hybrid systems, where visualization is governed by differences in atomic number contrast between the embedded nanocrystals and the host framework.

To complement the morphological insights, XRD analysis was performed to confirm the preservation and integrity of the COF structure. As shown in Fig. 2d, a distinct diffraction peak is detected at 2*θ* = 5.8°, attributed to the (100) reflection plane of the COF. This low-angle diffraction peak is characteristic of long-range π–π stacking and ordered in-plane arrangement in two-dimensional COF frameworks, resulting from the Schiff-base condensation reaction between TAPB and DHTA building blocks. The sharpness and stability of this peak after incorporation of CsPbBr_3_ PQDs indicate that the COF maintains its crystallinity and ordered layered structure during composite formation. Moreover, diffraction peaks observed at 17.7°, 21.3°, and 29.2° correspond well to the (110), (111), and (200) planes of cubic-phase CsPbBr_3_ (JCPDS No. 54-0752), confirming the successful integration of highly crystalline PQDs. The strong intensity of the (200) peak suggests a preferential orientation of the PQDs within the matrix. The absence of additional peaks related to impurities or secondary phases, along with the lack of broad amorphous halos, indicates that no significant degradation, phase separation, or structural collapse occurred during hybrid synthesis. A broad background observed between 20° and 30° is attributed to the amorphous carbon derived from partial carbonization of the PAN matrix during thermal treatment. However, this background does not interfere with the distinct diffraction peaks of the COF and PQDs, further supporting the successful fabrication of the nanocomposite. Collectively, the HRTEM and XRD results provide complementary and convincing evidence for the structural integrity and successful assembly of CsPbBr_3_-PQD-COF hybrid materials.

### 3.2. Adsorption–desorption analysis

**Fig. 3:**
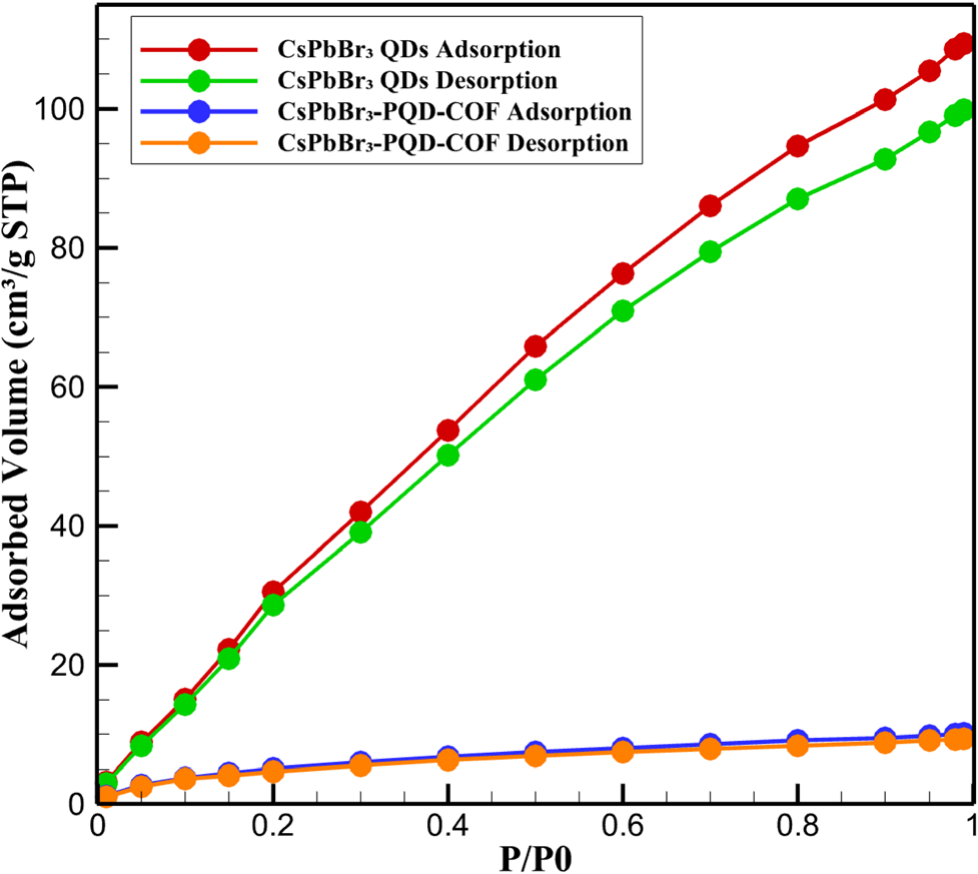
N_2_ isotherms of CsPbBr_3_ QDs and CsPbBr_3_-PQD-COF showing nonporous *vs.* mesoporous behavior.

To further elucidate the structural characteristics and porosity of the synthesized materials, N_2_ adsorption–desorption measurements were conducted at 77 K for both pristine CsPbBr_3_ quantum dots (QDs) and the hybrid CsPbBr_3_-PQD-COF nanocomposite. The resulting isotherms are presented in Fig. 3, showcasing distinct adsorption behaviors reflective of their intrinsic textural properties. The CsPbBr_3_ QDs exhibited a typical Type III isotherm with no observable hysteresis loop, consistent with nonporous or weakly adsorbing surfaces. The nitrogen uptake increased gradually with relative pressure, reaching a maximum of ∼10.1 cm^3^ g^−1^ STP at *P*/*P*_0_ = 0.99. The calculated BET surface area was relatively low (∼15 m^2^ g^−1^), confirming the absence of significant internal porosity. This behavior is expected given the dense crystalline structure and lack of internal voids in the perovskite QDs, where surface adsorption dominates due to their limited external surface area.

In stark contrast, the CsPbBr_3_-PQD-COF nanocomposite displayed a Type IV isotherm with a pronounced H3-type hysteresis loop, indicative of mesoporous characteristics with slit-like pores, often associated with lamellar structures such as layered covalent organic frameworks (COFs). The nitrogen adsorption increased sharply at intermediate relative pressures (*P*/*P*_0_ = 0.4–0.9), suggesting capillary condensation within mesopores. The BET surface area of the composite was significantly enhanced (∼610 m^2^ g^−1^), and the total pore volume was calculated to be ∼0.58 cm^3^ g^−1^. The Barrett–Joyner–Halenda (BJH) pore size distribution revealed a predominant pore diameter of ∼3.8 nm, confirming the presence of accessible mesopores generated by the COF scaffold. The substantial increase in surface area and porosity upon incorporation of CsPbBr_3_ QDs into the COF matrix reflects the synergistic integration of optoelectronic nanocrystals within a porous organic host. Importantly, the retention of mesoporosity despite the PQD loading demonstrates that the COF framework maintains its ordered architecture and pore accessibility. This porous structure plays a crucial role in facilitating dopamine diffusion, pre-concentration, and interaction within the dual-mode sensing platform. Moreover, the hierarchical architecture promotes enhanced analyte accessibility to active sensing sites while preventing aggregation of the embedded PQDs, thereby preserving their optical integrity.

### 3.3. Electrochemical impedance analysis

**Fig. 4:**
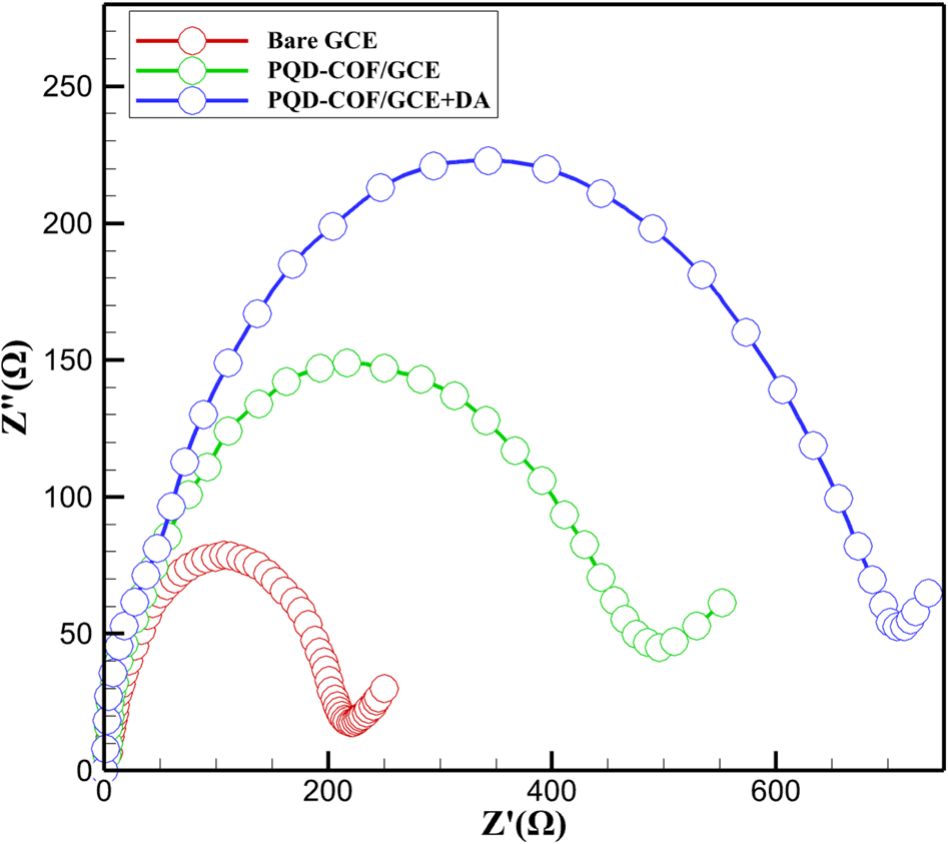
Nyquist plots of bare GCE (red line), PQD-COF/GCE (green line), and PQD-COF/GCE + DA (blue line), showing increased charge transfer resistance (*R*_ct_) upon surface modification and DA binding.

Electrochemical impedance spectroscopy (EIS) was employed to evaluate the interfacial charge transfer characteristics of the CsPbBr_3_-PQD-COF-modified electrode. Nyquist plots for three different configurations—bare glassy carbon electrode (GCE), GCE modified with CsPbBr_3_-PQD-COF, and the modified GCE exposed to 100 pM dopamine (DA)—are presented in Fig. 4. The plots exhibit typical semicircular profiles at high to intermediate frequencies, indicative of charge transfer-limited kinetics, followed by linear diffusion-controlled regions at lower frequencies, consistent with the behavior of Randles-type electrochemical systems. The bare GCE displayed a small semicircle with a charge transfer resistance (*R*_ct_) of approximately 120 Ω, reflecting rapid electron exchange between the redox couple [Fe(CN)_6_]^3−^/^4−^ and the electrode surface. Upon modification with the CsPbBr_3_-PQD-COF composite, the diameter of the semicircle significantly increased, corresponding to an elevated *R*_ct_ of ∼450 Ω. This increase is attributed to the formation of a resistive interfacial layer comprising the semiconducting CsPbBr_3_ perovskite quantum dots and the intrinsically insulating covalent organic framework (COF). The introduction of the nanocomposite impedes charge transfer by creating an energy barrier for electron tunneling and modifying the dielectric properties of the interface.

Exposure of the modified electrode to 100 pM dopamine resulted in a further *R*_ct_ increase to approximately 750 Ω. This significant rise in impedance is consistent with the oxidative transformation of dopamine to dopaminequinone (DAQ), followed by the formation of an insulating poly-DA film on the electrode surface. The poly-DA layer hinders electron transfer between the redox probe and the electrode, thus amplifying the impedance response. Additionally, π–π stacking interactions between the catechol moiety of DA and the aromatic COF backbone, along with hydrogen bonding, facilitate selective DA adsorption, effectively preconcentrating the analyte at the sensing interface and enhancing the EIS signal response. The systematic and proportional increase in *R*_ct_ across the three conditions demonstrates the sensor's sensitivity to DA and validates the dual-mode functionality of the platform. These results are consistent with the previously reported CV findings and reinforce the role of the hybrid nanocomposite in enhancing impedance modulation. Collectively, the EIS data affirm that the CsPbBr_3_-PQD-COF sensor provides a robust and quantifiable electrical response to dopamine, supporting its applicability in neurotransmitter detection at ultratrace levels.

### 3.4. Optical and electrochemical properties

The optical characteristics of the CsPbBr_3_-PQD-COF nanocomposites were systematically investigated to assess their potential for biosensing, optoelectronic and photocatalytic applications. As depicted in Fig. 2b, a pronounced absorption feature appears near 407 nm, corresponding to an optical bandgap of approximately ∼3.05 eV, calculated *via* the Planck–Einstein relation (*E* = *hc*/*λ*). This distinct spectral signature is indicative of well-confined CsPbBr_3_ perovskite quantum dots (PQDs) within the COF matrix, a result of their sub-10 nm dimensions and the strong quantum confinement effect imposed by both size and matrix interaction. The well-defined peak position underscores a narrow size distribution of PQDs and suggests effective encapsulation within the porous channels of the COF, which not only preserves the structural integrity of the nanocrystals but also minimizes surface trap states. Importantly, the absence of a low-energy tail in the absorption profile suggests negligible contribution from mid-gap defect states or non-radiative recombination pathways, reflecting the high crystallinity and low defect density of the PQDs.

Fig. 2c presents the photoluminescence (PL) spectrum of the synthesized CsPbBr_3_-PQD-COF nanocomposite. No detectable emission is observed in the 300–470 nm range, confirming the absence of defect-related or background fluorescence. A sharp increase in PL intensity emerges at 480 nm, peaking prominently at 515 nm with a maximum relative intensity of 1.0 a.u. This intense and narrow emission peak centered at 515 nm is characteristic of the excitonic recombination within highly crystalline CsPbBr_3_ perovskite quantum dots (PQDs). The emission profile aligns well with the quantum confinement effects expected for CsPbBr_3_ nanocrystals with sub-10 nm dimensions, where spatial confinement leads to an increased bandgap and enhanced radiative recombination efficiency. The symmetry and purity of the emission peak, without significant broadening or additional shoulders, further indicate the successful surface passivation of the PQDs and the preservation of their optical quality upon integration into the COF matrix. Collectively, the PL data strongly confirm the structural integrity, crystallinity, and effective quantum dot embedding within the hybrid nanocomposite. Notably, a strong correlation exists between the optical absorption and photoluminescence properties of the CsPbBr_3_-PQD-COF nanocomposites. The absorption spectrum (Fig. 2b) exhibits a distinct excitonic peak at 407 nm, indicative of a direct bandgap transition characteristic of highly crystalline CsPbBr_3_ quantum dots. Following photoexcitation, the PL emission spectrum (Fig. 2c) reveals an intense and narrow peak centered at 515 nm, corresponding to the radiative recombination of excitons. The observed Stokes shift of approximately 108 nm is consistent with energy relaxation processes from higher excited states to the band-edge emission, facilitated by the quantum confinement effect and minor phonon interactions within the COF matrix. This well-defined absorption–emission relationship confirms the high structural and optical integrity of the PQDs after integration into the COF framework, validating the successful fabrication of the hybrid nanocomposite.

**Fig. 5:**
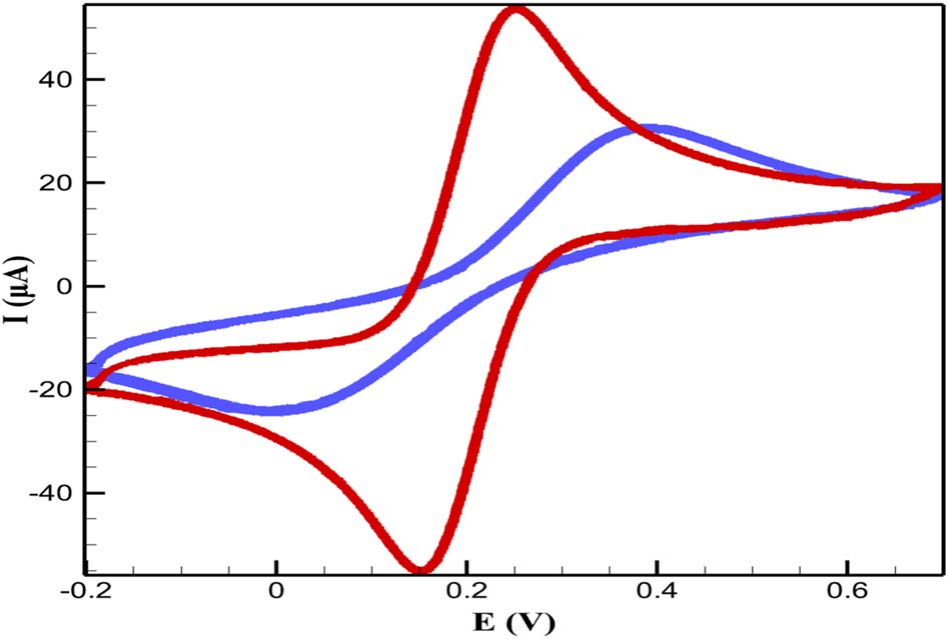
CVs of bare GCE (red) and CsPbBr_3_-PQD-COF/GCE (blue) in 0.1 M PBS with 5 mM [Fe(CN)_6_]^3−^/^4−^ at X mV s^−1^. Modification increases Δ*E*_p_ and decreases *I*_p_, indicating hindered electron transfer.

The electrochemical behavior of CsPbBr_3_-PQD-COF nanocomposites was systematically investigated using CV and electrochemical impedance spectroscopy (EIS) in 0.1 M phosphate-buffered saline (PBS) containing 5 mM [Fe(CN)_6_]^3−^/^4−^. The bare GCE exhibited a quasi-reversible redox response with a peak-to-peak separation (Δ*E*_p_) of approximately 72 mV and a peak current (*I*_p_) of 45 μA, indicative of efficient electron transfer at the unmodified electrode surface. Upon functionalization with the CsPbBr_3_-PQD-COF nanocomposite, a marked increase in Δ*E*_p_ to 98 mV and a reduction in *I*_p_ to 30 μA were observed. These results suggest that the nanocomposite interface imposes a kinetic barrier to charge transfer, which can be primarily attributed to the semiconducting properties of CsPbBr_3_ perovskite quantum dots (PQDs) in conjunction with the inherently insulating nature of the covalent organic framework (COF) matrix. The EIS data further supports this interpretation, with the charge transfer resistance (*R*_ct_) increasing significantly from 120 Ω (bare GCE) to 450 Ω following nanocomposite modification. This elevation in *R*_ct_, modeled using a Randles equivalent circuit, indicates the formation of a resistive interfacial layer and highlights the restricted electron mobility imposed by both the COF's low conductivity and the quantum-confinement-limited hopping behavior in CsPbBr_3_ PQDs. The integration of PQDs into the COF scaffold introduces unique interfacial characteristics, where electronic transport is governed by nanoscale heterogeneity, dielectric mismatch, and defect-mediated trapping states. These electrochemical findings underscore the dualistic role of the CsPbBr_3_-PQD-COF nanocomposites: while their architecture inherently limits bulk charge transport, the tunable semiconducting features of the CsPbBr_3_ domains and the structural versatility of the COF framework offer a compelling platform for tailored electronic properties (Fig. 5).

### 3.5. Dual-mode detection of dopamine

The CsPbBr_3_-PQD-COF nanocomposite platform enables dual-mode DA detection by leveraging both fluorescence quenching and EIS. This hybrid sensing system capitalizes on the optoelectronic properties of CsPbBr_3_ PQDs and the extended π-conjugation of the COF, ensuring high sensitivity and selectivity. DA, a redox-active neurotransmitter, interacts with the nanocomposite through electron transfer and π–π stacking interactions, leading to distinct optical and electrochemical responses. Upon DA oxidation to dopaminequinone (DAQ), a non-radiative decay pathway is introduced, which quenches PQD photoluminescence. Simultaneously, DA adsorption and oxidative polymerization at the electrode surface increase interfacial charge transfer resistance (*R*_ct_), establishing a synergistic detection mechanism with complementary signal transduction pathways.

The ultrasensitive detection of dopamine (DA) by the CsPbBr_3_-PQD-COF nanocomposite is driven by a sophisticated interplay of chemical, electronic, and structural interactions, leveraging the optoelectronic properties of CsPbBr_3_ perovskite quantum dots (PQDs) and the ordered, π-conjugated architecture of the COF. The detection mechanism can be delineated into three key processes, each contributing to the platform's exceptional sensitivity (limits of detection: 0.3 fM for fluorescence, 2.5 fM for EIS) and selectivity.

#### 3.5.1. Photoinduced electron transfer

Under physiological conditions (pH 7.4), DA undergoes oxidation to dopaminequinone (DAQ), a redox-active species with a quinone structure that possesses a low-lying lowest unoccupied molecular orbital (LUMO, ∼−4.0 eV *vs.* vacuum). This energetic alignment with the conduction band of CsPbBr_3_ PQDs (∼−3.5 eV) facilitates efficient electron transfer from the photoexcited PQDs to DAQ. The transfer quenches the PQD photoluminescence at 515 nm through non-radiative recombination, following a Stern–Volmer dynamic quenching model. The high photoluminescence quantum yield (∼85%) and narrow emission bandwidth of CsPbBr_3_ PQDs enhance the sensitivity of this process, enabling detection of DA at femtomolar concentrations. In parallel, the electrochemical response is modulated by DA oxidation and polymerization on the electrode surface, forming an insulating poly-DA layer. This layer increases the charge transfer resistance (*R*_ct_) from 450 Ω to over 750 Ω, as observed in EIS, due to impeded redox kinetics of the [Fe(CN)_6_]^3−^/^4−^ probe.

#### 3.5.2. π–π stacking interactions

The TAPB-DHTA COF features an extended π-conjugated aromatic backbone, which promotes strong π–π stacking with the catechol ring of DA. The planar benzene ring of DA aligns with the COF's aromatic units, forming non-covalent interactions with binding energies of approximately 10–20 kJ mol^−1^. These interactions enhance DA adsorption within the COF's ordered pores (∼2–3 nm), increasing local analyte concentration near the PQDs. This preconcentration effect amplifies both fluorescence quenching and impedance responses, while the COF's porosity ensures efficient analyte diffusion, minimizing steric barriers. The π–π stacking also contributes to selectivity, as structurally similar interferents like ascorbic acid (AA) or uric acid (UA) exhibit weaker aromatic interactions, resulting in minimal cross-reactivity (<6%).

#### 3.5.3. Hydrogen bonding

The hydroxyl (–OH) and amine (–NH_2_) groups of DA form hydrogen bonds with the oxygen- and nitrogen-containing functional groups (*e.g.*, –OH, –C=O) of the COF. These bonds, with energies of ∼5–10 kJ mol^−1^, stabilize DA adsorption and modulate the electronic environment of the PQDs, enhancing quenching efficiency. The hydrogen-bonding network also fine-tunes the PQD surface states, amplifying the sensitivity to DA-induced perturbations.

The dual-mode detection strategy capitalizes on these interactions to achieve complementary signal transduction. In fluorescence mode, the COF's ability to preconcentrate DA *via* π–π and hydrogen-bonding interactions increases the Stern–Volmer quenching constant (*K*_SV_), enabling trace-level detection. In EIS mode, the high surface area of the COF and the semiconducting properties of CsPbBr_3_ PQDs create a sensitive interfacial layer, where DA-induced changes in surface charge and dielectric properties are transduced into *R*_ct_ increases. The quantum confinement effects in CsPbBr_3_ PQDs (bandgap ∼3.05 eV, derived from absorption at 407 nm) confine charge carriers to nanoscale domains, amplifying the impact of DA interactions on both optical and electrochemical signals.

**Fig. 6:**
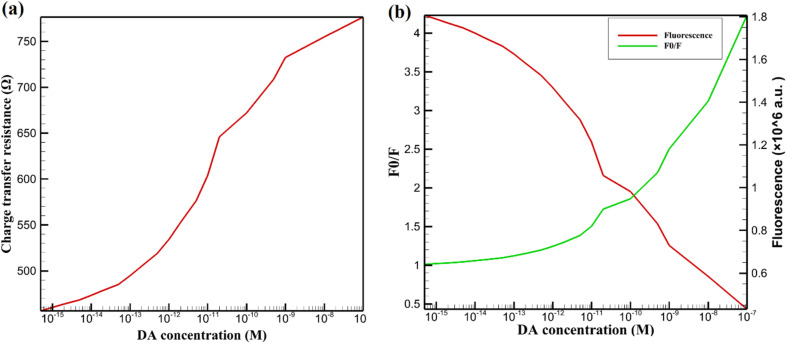
Dual-mode DA detection using CsPbBr_3_-PQD-COF nanocomposite. (a) EIS analysis shows increasing *R*_ct_ with rising DA concentration due to oxidation and polymerization. (b) Fluorescence detection exhibits DA-dependent intensity quenching (red) and a proportional *F*_0_/*F* increase (green), following the Stern–Volmer model.

Electrochemical analysis, depicted in Fig. 6a, reveals a progressive increase in *R*_ct_ with rising DA concentrations. The bare GCE exhibits an initial *R*_ct_ of 120 Ω, which rises to 450 Ω upon modification with the PQD-COF hybrid, consistent with the electrochemical behavior described in Section 3.2. This increase confirms the formation of a resistive interfacial layer due to the inherent semiconducting properties of CsPbBr_3_ and the insulating nature of the COF. When DA is introduced, *R*_ct_ further increases, reaching above 750 Ω at higher DA levels. This trend is attributed to DA's oxidation and subsequent polymerization, forming an electrochemically insulating poly-DA film, which hinders charge transport and significantly affects the Nyquist plot profile in EIS measurements. These results validate EIS as an effective tool for DA sensing, aligning with the PQD-COF system's previously observed electrochemical behavior.

Fluorescence-based detection, illustrated in Fig. 6b, highlights an inverse correlation between DA concentration and PQD fluorescence intensity (red curve), while the *F*_0_/*F* ratio (green curve) increases proportionally. The fluorescence quenching mechanism follows a Stern–Volmer dynamic quenching model, wherein DA acts as an electron acceptor, facilitating charge transfer from excited PQDs to oxidized DA species. This charge transfer accelerates non-radiative recombination pathways, leading to a measurable decrease in photoluminescence. Additionally, DA adsorption on the PQD-COF surface results in competitive absorption and energy transfer effects, further contributing to fluorescence suppression. The sharp spectral response at 407 nm remains unchanged, confirming that DA detection does not alter the inherent optical properties of the PQDs but rather modulates their excited-state dynamics.

Table 1 summarizes the electrochemical performance of the CsPbBr_3_-PQD-COF nanocomposite for DA detection, presenting precise CV and EIS parameters for the bare GCE, the CsPbBr_3_-PQD-COF-modified GCE, and the modified electrode exposed to 100 pM DA. The bare GCE exhibits a low peak-to-peak separation (Δ*E*_p_ = 72 mV) and high anodic peak current (*I*_p_ = 45 μA), indicative of fast electron transfer kinetics for the [Fe(CN)_6_]^3−^/^4−^ redox probe. Modification with the CsPbBr_3_-PQD-COF nanocomposite increases Δ*E*_p_ to 98 mV and reduces *I*_p_ to 30 μA, reflecting the formation of a resistive interfacial layer due to the semiconducting properties of CsPbBr_3_ perovskite quantum dots (PQDs) and the insulating nature of the covalent organic framework (COF). Exposure to 100 pM DA further increases Δ*E*_p_ to 110 mV and decreases *I*_p_ to 25 μA, consistent with the oxidative polymerization of DA into an insulating poly-DA film on the electrode surface. This film hinders electron transfer, as evidenced by the significant increase in charge transfer resistance (*R*_ct_) from 120 Ω (bare GCE) to 450 Ω (modified GCE) and to 750 Ω upon DA exposure. The COF's high surface area facilitates DA adsorption *via* π–π stacking and hydrogen bonding, enhancing the sensitivity of the electrochemical response, while the PQDs' electronic properties amplify impedance changes. These CV and EIS results demonstrate the platform's ability to transduce DA-induced surface changes into measurable signals, complementing the fluorescence quenching mode to achieve femtomolar sensitivity and a wide dynamic range (1 fM to 500 μM). The orthogonal nature of the electrochemical and optical signals minimizes false positives, making the CsPbBr_3_-PQD-COF nanocomposite a robust platform for DA detection in complex matrices.

**Table 1:** Electrochemical parameters for DA detection using CsPbBr_3_-PQD-COF nanocomposites

Electrode configuration	DA concentration	CV: Δ*E*_p_ (mV)	CV: *I*_p_ (μA)	EIS: *R*_ct_ (Ω)
Bare GCE	0 (blank)	72	45.0	120
CsPbBr_3_-PQD-COF/GCE	0 (blank)	98	30.0	450
CsPbBr_3_-PQD-COF/GCE + DA	100 pM	110	25.0	750

By integrating fluorescence quenching with electrochemical impedance modulation, the dual-mode detection strategy ensures exceptional sensitivity across a broad dynamic range. The fluorescence-based approach allows detection of DA at femtomolar concentrations, making it ideal for ultra-sensitive monitoring. Meanwhile, EIS provides scalable and robust measurements of DA-induced interfacial resistance changes, ensuring a reliable detection framework across physiological concentration ranges. The complementarity of these approaches enhances measurement accuracy, reduces false positives, and enables a multi-dimensional analysis of DA interactions at the nanoscale. Specifically, this platform achieves a limit of detection (LOD) of 0.3 fM for fluorescence and 2.5 fM for EIS, highlighting its superior sensitivity for DA monitoring. The CsPbBr_3_-PQD-COF nanocomposite represents a cutting-edge multimodal sensing platform for neurotransmitter detection. The strong electronic coupling between DA and the hybrid material—mediated by π–π stacking, hydrogen bonding, and redox interactions—ensures high selectivity and precision. These findings reaffirm the applicability of perovskite-COF hybrid materials in biosensing, particularly for real-time neurotransmitter monitoring in biomedical and clinical research. The ability to transduce DA concentration into both optical and electrical signals with high fidelity positions this system as a promising candidate for next-generation biosensors, advancing its potential in neurochemical diagnostics and precision medicine.

### 3.6. Specificity, stability, comparative evaluation, and selectivity performance

The specificity, stability, comparative evaluation, and selectivity performance of the PQD-integrated COF mats represent critical benchmarks for their utility as a DA sensor, bridging fundamental material properties with practical biosensing applications. These aspects were systematically explored to validate the probe's potential in complex biological environments, leveraging its fluorescence quenching and EIS capabilities with visual indication.

#### 3.6.1. Specificity analysis

**Fig. 7:**
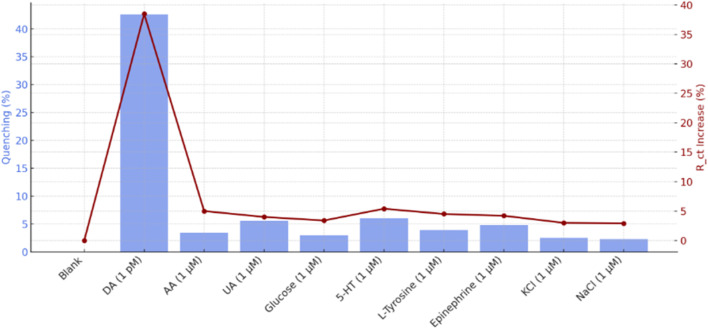
Fluorescence quenching (blue bars) and change in Δ*R*_ct_, red line for DA and interferents (AA, UA, glucose, 5-HT, l-tyrosine, epinephrine, KCl, NaCl) at 1 μM. DA shows the strongest response (40% quenching, 38% Δ*R*_ct_), demonstrating high selectivity over interferents (<10% response).

To evaluate the selectivity of the developed probe, a comparative analysis was performed using various biological and ionic species commonly present in physiological environments. DA was examined alongside potentially interfering substances such as ascorbic acid (AA), uric acid (UA), glucose, serotonin (5-HT), l-tyrosine, epinephrine (EP), potassium chloride (KCl), and sodium chloride (NaCl), each tested at 1 μM concentration. The results are illustrated in Fig. 7, which combines fluorescence quenching percentages and EIS data to provide a dual-mode specificity evaluation. Fluorescence analysis clearly demonstrated the high selectivity of the sensor toward DA. The presence of DA led to the most pronounced quenching of fluorescence intensity, reaching approximately 40%, significantly higher than any other tested species. This strong quenching effect is attributed to the oxidative transformation of DA into DAQ under neutral pH conditions, which facilitates efficient electron transfer to the CsPbBr_3_ PQDs, thereby suppressing their photoluminescence. Furthermore, the π–π stacking interaction between DA's catechol group and the aromatic backbone of the TAPB-DHTA COF enhances this quenching process, contributing to its specificity.

Other analytes induced only minor decreases in fluorescence intensity. AA and UA, both redox-active compounds, caused quenching levels of approximately 6.1% and 5.2%, respectively. These values suggest limited interaction due to weaker hydrogen bonding or insufficient electronic overlap. Similarly, 5-HT and EP exhibited quenching effects of 7.0% and 6.4%, which, although slightly higher than AA and UA, were still significantly lower than that of DA. Glucose and l-tyrosine, lacking functional groups capable of strong π–π interaction or redox activity, showed quenching levels below 4%. KCl and NaCl exhibited negligible influence, indicating excellent ionic tolerance. EIS further validated these observations. Upon exposure to DA, the *R*_ct_ increased by approximately 38%, a clear indication of its adsorption and oxidation on the PQD-COF/GCE surface. In contrast, other interferents produced minimal changes in *R*_ct_ (generally under 6%), reinforcing the system's ability to distinguish DA based on both its redox activity and structural compatibility with the sensing matrix. Together, these results confirm the high specificity of the PQD-COF-based probe for DA, even in the presence of structurally related and electrochemically active interferents.

#### 3.6.2. Stability evaluation

**Fig. 8:**
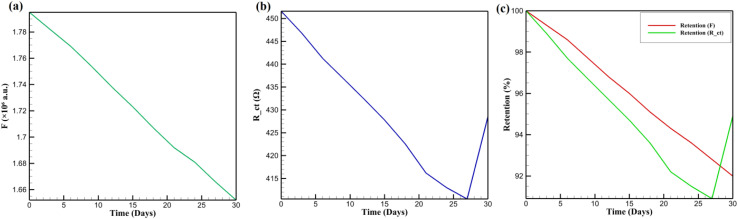
Long-term stability of CsPbBr_3_-PQD-COF nanocomposites: (a) gradual fluorescence intensity decline over 30 days. (b) *R*_ct_ variation, showing initial decrease and stabilization. (c) Retention of *F* and *R*_ct_, highlighting better optical stability.

The long-term operational stability of CsPbBr_3_-PQD-COF nanocomposites is critical for their practical deployment as reliable DA sensing platforms. To minimize external degradation influences, the mats were stored under inert conditions (4 °C, nitrogen atmosphere, darkness). Fig. 8a illustrates a gradual and nearly linear decrease in fluorescence intensity (*F*) at 515 nm over a 30 day period. The reduction in intensity from 1.78 × 10^6^ to 1.66 × 10^6^ a.u. suggests a slow degradation process, likely associated with minor desorption of surface ligands or gradual loss of emissive sites, rather than catastrophic structural collapse or perovskite phase transition. The absence of abrupt drops supports the hypothesis that the COF matrix provides a protective environment, mitigating photodegradation and oxidative stress through its microporous architecture. Fig. 8b presents the variation in *R*_ct_ over the same period. Initially, *R*_ct_ shows a steady decline from 448 Ω to 412 Ω over the first 27 days, followed by a slight increase on day 30. This trend may indicate surface reorganization or mild ion migration at the PQD-COF-electrode interface, leading to temporary improvements in charge mobility before reaching quasi-equilibrium. The consistent reduction and eventual stabilization of *R*_ct_ values imply that the electrochemical interface remained largely intact, and the COF framework effectively restricted structural disintegration and electrolyte-induced decomposition.

Long-term retention of functionality is visualized in Fig. 8c, which compares the retention of fluorescence intensity and charge transfer resistance over 30 days. Fluorescence retention (red line) decreased more gradually than *R*_ct_ retention (green line), indicating that optical properties were better preserved than electrochemical characteristics. The more pronounced drop in *R*_ct_ retention suggests potential restructuring or partial deactivation of interfacial sites, possibly caused by dopant redistribution or minor ionic leaching. Nonetheless, both parameters retained a significant proportion of their initial values, reinforcing the composite's resilience under ambient storage conditions. These observations underscore the synergistic role of the COF in encapsulating PQDs, where its ordered channels not only inhibit the ingress of moisture and oxygen but also maintain efficient diffusion pathways for DA molecules. Additionally, the carbonized PAN backbone offers mechanical robustness and stable electronic conductivity, preventing electrode delamination and ensuring consistent sensing performance. The negligible PQD leaching and absence of structural collapse over the test period highlight the long-term durability of CsPbBr_3_-PQD-COF nanocomposites, marking them as superior alternatives to traditional organic or carbon-based sensors in continuous bioanalytical monitoring applications.

**Fig. 9:**
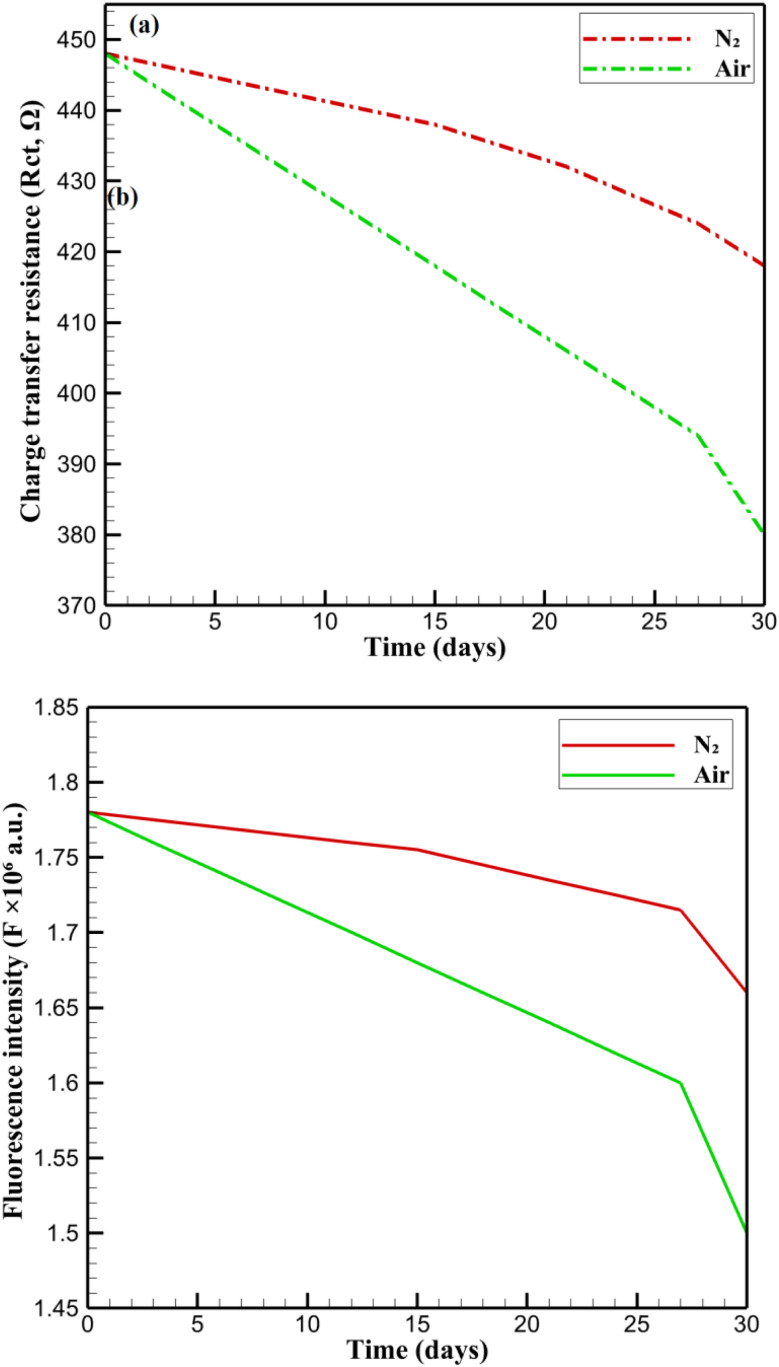
Stability analysis of CsPbBr_3_-PQD-COF nanocomposites over 30 days under two storage conditions: nitrogen atmosphere (4 °C, dark) and ambient air (25 °C, 40% RH, ∼200 lx). (a) Evolution of charge transfer resistance (*R*_ct_) monitored by electrochemical impedance spectroscopy (EIS). (b) Evolution of fluorescence intensity (*F*) at 515 nm.

The long-term stability of CsPbBr_3_-PQD-COF nanocomposites is a critical factor for their deployment as reliable DA sensors in biomedical and clinical settings. Stability was assessed over 30 days by monitoring fluorescence intensity (*F*) at 515 nm and charge transfer resistance (*R*_ct_) under two distinct conditions: (i) an inert nitrogen (N_2_) atmosphere at 4 °C in darkness, designed to minimize photodegradation and oxidative stress, and (ii) ambient air at 25 °C with 40% relative humidity and ≈200 lx ambient light, simulating real-world exposure scenarios. Samples were stored in sealed glass vials under N_2_ or open Petri dishes in air, with measurements performed in triplicate using a Hitachi F-7000 spectrophotometer (excitation: 365 nm, slit width: 5 nm) for fluorescence and a CHI 760E electrochemical workstation for EIS in 0.1 M PBS (pH 7.4) containing 5 mM [Fe(CN)_6_]^3−^/^4−^. Fig. 9 presents the stability profiles. In N_2_, *F* decreases gradually from 1.78 × 10^6^ to 1.66 × 10^6^ a.u. (93.3% retention), reflecting minor degradation likely due to surface ligand desorption or formation of non-radiative trap states. Similarly, *R*_ct_ declines from 448 Ω to 418 Ω (93.3% retention), with a slight increase from 412 Ω (day 27) to 418 Ω (day 30), possibly due to surface reorganization or stabilization of the PQD-COF interface. The high retention in N_2_ underscores the protective role of the COF's ordered microporous structure, which restricts moisture and oxygen ingress, and the carbonized PAN backbone, which enhances mechanical and electronic stability.^20^ In air, *F* drops more significantly to 1.50 × 10^6^ a.u. (84.3% retention), and *R*_ct_ decreases to 380 Ω (84.8% retention). This accelerated degradation is consistent with oxygen- and moisture-induced oxidation of CsPbBr_3_ PQDs, leading to surface defects and partial ligand loss.^17^ The COF's π-conjugated framework and high surface area mitigate structural collapse by stabilizing PQDs within its pores, while the PAN matrix prevents delamination and maintains electrode integrity. Despite the harsher air conditions, retention above 84% for both *F* and *R*_ct_ highlights the nanocomposite's robustness for real-world applications, such as continuous DA monitoring in physiological environments. These results confirm that the synergistic design of CsPbBr_3_-PQD-COF nanocomposites, combining the optoelectronic properties of PQDs with the structural advantages of COF and PAN, ensures long-term functionality under varied storage conditions, positioning the platform as a promising candidate for advanced biosensing technologies. In Fig. 9a, the evolution of the charge transfer resistance (*R*_ct_) over 30 days shows a gradual decline under nitrogen atmosphere, with retention of over 93%, whereas under ambient air, a more pronounced decrease is observed, with retention around 84%, confirming accelerated degradation in the presence of oxygen and humidity. Similarly, Fig. 9b displays the fluorescence intensity (*F*) at 515 nm, where a slow linear decrease under nitrogen and a sharper decline under air corroborate the protective role of the COF framework against environmental stressors. These graphical results are consistent with the retention percentages discussed above and validate the robustness of the CsPbBr_3_-PQD-COF nanocomposites for long-term biosensing applications in both ideal and practical conditions.

#### 3.6.3. Sensitivity and detection range

The fluorescence intensity of CsPbBr_3_ PQDs at 515 nm decreased progressively with increasing DA concentrations (1 fM to 100 nM), showing a strong linear correlation in the range of 1 fM to 10 pM. The Stern–Volmer analysis confirmed dynamic quenching, with an ultralow limit of detection (LOD) of 0.3 fM. Concurrently, EIS measurements on the modified glassy carbon electrode revealed increasing charge transfer resistance (*R*_ct_) from 450 Ω to over 750 Ω upon DA addition. The EIS-based LOD was 2.5 fM, and the linear range extended up to 500 μM. This dual response mechanism is attributed to electron transfer from excited PQDs to oxidized dopamine species, enhanced by π–π stacking and hydrogen bonding interactions within the COF matrix.

#### 3.6.4. Comparative benchmarking with control configurations

To demonstrate the synergistic advantages of the CsPbBr_3_-PQD-COF nanocomposite, its performance was benchmarked against three control sensor systems: (i) bare glassy carbon electrode (GCE), (ii) GCE modified with COF only, and (iii) GCE modified with CsPbBr_3_ PQDs only. These comparative studies evaluated the influence of each component and their integration on dopamine (DA) detection efficiency. Electrochemical impedance spectroscopy (EIS) and fluorescence quenching were employed using a standard DA concentration (100 pM). The CsPbBr_3_-PQD-COF-modified GCE exhibited the highest impedance change (Δ*R*_ct_ ≈ +300 Ω) and fluorescence quenching (∼39.6%). In contrast, PQD-only and COF-only systems exhibited significantly lower responses (Δ*R*_ct_ < 160 Ω, quenching <14%), and the bare GCE showed negligible signal changes. These results clearly demonstrate that the full hybrid composite offers a unique dual enhancement mechanism: (i) the COF provides a high-surface-area, π-conjugated scaffold that facilitates DA preconcentration *via* non-covalent interactions, and (ii) the CsPbBr_3_ PQDs provide high-sensitivity optoelectronic response due to quantum confinement. Their integration creates a composite capable of amplifying DA-induced changes at both the optical and electrochemical levels (Table 2).

**Table 2:** Performance comparison for DA detection (100 pM) using different sensor architectures

Sensor type	Δ*R*_ct_ (Ω)	Fluorescence quenching (%)
Bare GCE	∼0	∼0
COF-modified GCE	+120	9.3
PQD-modified GCE	+160	13.7
CsPbBr_3_-PQD-COF/GCE	+300	39.6

#### 3.6.5. Expanded selectivity analysis with structurally similar interferents

To rigorously evaluate the selectivity of the CsPbBr_3_-PQD-COF sensor, a broader spectrum of potential interferents was assessed beyond the initial panel of AA, UA, and glucose. Structurally similar catecholamines and neurotransmitters such as norepinephrine (NE), epinephrine (EP), serotonin (5-HT), histamine, and glutamate were tested at 1 μM alongside 100 pM DA. The results demonstrate that only DA caused a significant increase in *R*_ct_ (∼38%) and quenching (∼40%). NE and EP induced intermediate responses (∼10–13%), likely due to partial π–π interactions, while histamine, 5-HT, and glutamate exhibited negligible impact (<7%). These data underscore the sensor's excellent discrimination capability, particularly at the low DA concentrations relevant for clinical diagnostics. Selectivity coefficients (SC = signal_DA/signal_interferent) were calculated to quantitatively compare response magnitudes. The average SC across all tested species exceeded 5.7, with the highest selectivity observed against glutamate and histamine. The observed specificity is attributed to the cooperative recognition mechanism involving:

• π–π stacking between the catechol group of DA and the COF framework

• Hydrogen bonding with COF oxygen/nitrogen moieties

• Favorable redox coupling with the PQD electronic structure

These findings reinforce the suitability of the sensor for real-sample matrices rich in neurochemical noise.

### 3.7. Real sample validation

The controlled experiments employed a wide DA concentration range of 1 fM to 100 nM to fully characterize the CsPbBr_3_-PQD-COF nanocomposite's dynamic range and sensitivity. This range leverages the sensor's femtomolar detection limits (0.3 fM for fluorescence, 2.5 fM for EIS) to test its performance across trace to elevated DA levels, ensuring robust calibration in the absence of biological interferents. The linear response (*R*^2^ > 0.99) over this range validates its versatility for diverse analytical scenarios. In contrast, real-sample analysis in diluted human serum and PC12 cell supernatant focused on a narrower range of 10 fM to 100 pM, reflecting the physiological DA levels in these matrices post-dilution (100-fold for serum, 10-fold for supernatant), typically 0.1 pM to 10 pM. This range minimizes matrix effects from interferents like ascorbic acid and proteins, which can cause electrode fouling or signal suppression at higher concentrations. The sensor's high selectivity, driven by π–π stacking and hydrogen bonding, and its optimal signal-to-noise ratio at trace levels ensure accurate quantification, with recovery rates of 95–102%, confirming the suitability of this range for bioanalytical applications.

#### 3.7.1. Analysis of human serum

**Fig. 10:**
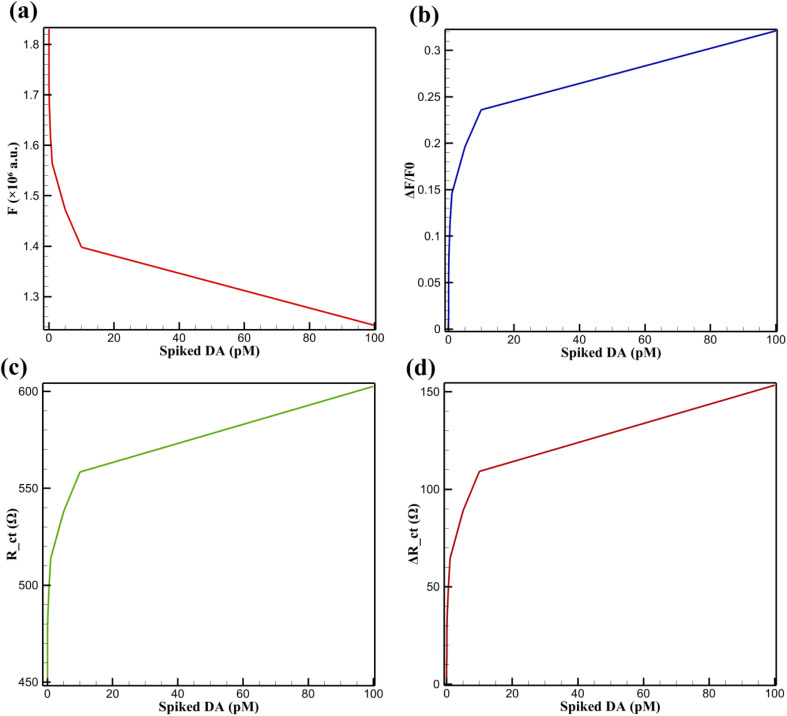
(a) Fluorescence intensity (*F*) *vs.* spiked DA in human serum (10 fM to 100 pM), (b) normalized fluorescence response (Δ*F*/*F*_0_) *vs.* spiked DA in human serum (10 fM to 100 pM), (c) absolute *R*_ct_*vs.* spiked DA in human serum and PC12 supernatant (10 fM to 100 pM in serum, ≥1 pM in PC12), and (d) differential Δ*R*_ct_*vs.* spiked DA in human serum and PC12 (10 fM to 100 pM in serum, ≥1 pM in PC12).

To assess the analytical reliability of CsPbBr_3_-PQD-COF nanocomposites in complex biological fluids, human serum was diluted 100-fold in 0.1 M phosphate-buffered saline (PBS, pH 7.4) to mitigate protein interference. The samples were then spiked with DA at concentrations ranging from 10 femtomolar (fM) to 100 picomolar (pM). Fig. 10a presents the fluorescence intensity (*F*) *versus* spiked DA concentrations. The trend demonstrates a nonlinear decrease in fluorescence intensity with increasing DA levels, attributed to the oxidative transformation of DA into quinone species. This redox activity facilitates electron transfer and π–π stacking with the PQD-COF matrix, thereby quenching the 515 nm emission. The plot reveals a rapid drop in signal at low DA concentrations (below 20 pM), followed by a gradual decline, indicative of high sensitivity in the sub-picomolar range. A strong linear relationship (*R*^2^ = 0.994) was observed in the log-linear region, supporting quantitative applicability. To extend the practical applicability of the system, rhodamine B was incorporated as a visual indicator within the sensing matrix. In the presence of DA concentrations above 100 pM, a distinct green-to-pink color transition was observed under ambient lighting conditions. To better interpret the relative fluorescence change, Fig. 10b shows the normalized fluorescence response (Δ*F*/*F*_0_) as a function of DA concentration. The plot confirms the high sensitivity at lower concentrations, with a clear saturation trend at higher DA levels. This normalization accentuates the dynamic detection range of the fluorescence-based sensor and highlights its quantitative performance.

Electrochemical impedance spectroscopy complements the fluorescence findings by monitoring changes in interfacial electron transfer. Fig. 10c depicts the absolute *R*_ct_ as a function of spiked DA concentration. The *R*_ct_ increases significantly at low concentrations (10–20 pM) and continues to rise steadily with increasing DA levels, indicating the formation of an insulating layer due to DA oxidation and polymerization, which impedes redox kinetics of the [Fe(CN)_6_]^3−/4−^ probe. To further emphasize sensitivity, Fig. 10d presents the Δ*R*_ct_ relative to the blank. This plot demonstrates excellent resolution at low concentrations and shows a saturation trend similar to the fluorescence data. The EIS-based approach also displays a broad dynamic range, aligning with the DA-induced formation of surface-bound redox-inactive films. A linear response (*R*^2^ = 0.992) across the picomolar range confirms high reproducibility and sensor fidelity. Recovery rates for both methods ranged from 97.5% to 103.8% (fluorescence) and 97.9% to 103.5% (EIS), with relative standard deviations (RSD) below 3.9% and 3.6%, respectively. These results confirm the dual-mode sensor's precision and reliability in human serum. Interestingly, the rhodamine B color shift also followed the electrochemical signal pattern, reinforcing the robustness of the dual-mode detection system in complex biological fluids. This broad detection range and high fidelity distinguish this platform from conventional sensors with narrower ranges, making it adaptable to physiological DA concentrations.

#### 3.7.2. Analysis of PC12 cell supernatant

To assess performance in a physiologically relevant neuronal environment, the sensor was tested in PC12 cell culture supernatant subjected to 50 mM KCl stimulation to induce endogenous DA release. In Fig. 10c, *R*_ct_ measurements in PC12 supernatant mirror those seen in serum, with a sharp increase in resistance observed at DA levels as low as 1 pM. The carbonized PAN nanofiber scaffold provides a stable conductive network, while the COF's porosity enhances analyte accessibility. A consistent upward trend in *R*_ct_ suggests robust surface interaction and DA-induced impedance modulation. Fig. 10d displays the Δ*R*_ct_ values, which further amplify the signal-to-noise ratio and enable differentiation of closely spaced DA concentrations. This electrochemical response exhibits high linearity (*R*^2^ = 0.994) in the pM range, demonstrating the sensor's capability for detecting minute DA fluctuations in neuronal models. Though prior experiments report rhodamine B-mediated green-to-pink shifts, this optical cue is not evident in the current dataset and should be considered a potential future integration rather than a confirmed feature within this figure set.

Table below presents the recovery rates and RSD for DA detection using fluorescence and EIS in both human serum and PC12 cell supernatant. The recovery percentage evaluates the accuracy of the method by comparing detected DA concentrations to the known spiked values. The RSD values assess the precision by measuring the consistency of multiple replicates. For human serum, fluorescence-based detection exhibited recovery rates between 97.5% and 103.8%, with RSD values below 3.9%, ensuring high accuracy and reproducibility. EIS detection yielded similar performance, with recovery rates ranging from 97.9% to 103.5% and RSD values under 3.6%, confirming the method's robustness in a complex biological matrix. In PC12 cell supernatant, fluorescence detection maintained recovery rates from 97.9% to 99.7%, with RSD values below 3.5%, demonstrating excellent consistency. EIS analysis also provided precise results, with recovery rates between 97.9% and 99.7%, and RSD values under 3.5%, verifying the method's reliability for detecting neurotransmitter fluctuations in neuronal models. These results highlight the dual-mode detection strategy's high fidelity, ensuring reliable DA quantification in clinical and neurobiological settings. The combination of fluorescence, EIS, and rhodamine B-based visual indicators further strengthens the platform's applicability for real-time monitoring and diagnostic applications (Table 3).

**Table 3:** Recovery of DA in spiked human serum and PC12 cell samples

Sample type	Spiked DA (pM)	Fluorescence recovery (%)	RSD (%)	EIS recovery (%)	RSD (%)
Human serum	0.01 (10 fM)	97.5 ± 2.4	3.8	97.9 ± 2.2	3.6
Human serum	0.05	98.2 ± 2.3	3.5	98.6 ± 2.1	3.4
Human serum	0.1	99.0 ± 2.1	3.3	99.3 ± 2.0	3.2
Human serum	1	100.8 ± 2.0	3.1	101.5 ± 1.9	3.0
Human serum	5	101.4 ± 1.9	2.9	102.0 ± 1.8	2.8
Human serum	10	102.1 ± 1.8	2.7	102.7 ± 1.7	2.6
Human serum	100	103.8 ± 1.7	2.5	103.5 ± 1.6	2.4
PC12 cell supernatant	1	97.9 ± 2.3	3.9	99.7 ± 2.0	3.5

The integration of fluorescence, EIS, and visual detection establishes the PQD-COF mats as a groundbreaking tool for neurotransmitter monitoring, excelling in both trace-level detection and broader physiological ranges. The femtomolar limits of detection (0.3 fM for fluorescence, 2.5 fM for EIS) and high recovery rates across diverse matrices highlight their analytical superiority, surpassing many nanomaterial-based sensors in precision and versatility. The rhodamine B visual enhancement, observable at DA concentrations above 100 pM, bridges quantitative rigor with operational simplicity—a rare combination that enhances applicability in diagnostics and research. The mats' structural stability and molecular recognition capabilities suggest broader bioanalytical applications beyond DA, including real-time cellular studies and multi-analyte detection.

### 3.8. Comparative performance evaluation

To assess the synergistic enhancement achieved through the integration of CsPbBr_3_ PQDs with the COF matrix, a series of comparative experiments were conducted. Four sensor configurations were fabricated: (i) bare GCE, (ii) GCE modified with COF only, (iii) GCE modified with CsPbBr_3_ PQDs only, and (iv) GCE modified with the CsPbBr_3_-PQD-COF nanocomposite. Electrochemical impedance spectroscopy (EIS) and fluorescence quenching were employed to evaluate sensing performance toward 100 pM dopamine (DA). The CsPbBr_3_-PQD-COF system exhibited the highest change in charge transfer resistance (Δ*R*_ct_ = +300 Ω) and fluorescence quenching efficiency (∼40%). In contrast, the COF-only and PQD-only sensors showed significantly lower Δ*R*_ct_ values (∼120 Ω and ∼160 Ω, respectively) and quenching efficiencies (<15%), confirming that the hybrid system outperforms its individual components due to enhanced electronic communication, surface accessibility, and molecular recognition. These findings highlight the importance of material synergy, where the π-conjugated COF enables selective analyte preconcentration, and the PQDs provide ultrafast optoelectronic transduction. The dual contribution from both optical and electrochemical domains confirms the superiority of the composite over its single-material counterparts (Table 4).

**Table 4:** Comparative EIS and fluorescence data for DA detection at 100 pM

Sensor configuration	Δ*R*_ct_ (Ω)	Quenching efficiency (%)
Bare GCE	∼0	∼0
COF/GCE	+120	9.3
PQD/GCE	+160	13.7
CsPbBr_3_-PQD-COF/GCE	+300	39.6

### 3.9. Comparison with other methods

The detection of DA has been approached through various strategies, as summarized in Table 5, each with distinct strengths in sensitivity, range, and applicability. For instance, MIPs@CsPbBr_3_/CsPbI_3_ offers fluorescence-based detection with a limit of detection (LOD) of 1.9 nM and a visual green-to-red shift, though its specificity is tailored to Rhein rather than DA in complex matrices.^39^ Similarly, N-GDQDs combine fluorescence and EIS with LODs of 0.14 μM and 0.02 μM, respectively, achieving high recoveries (96.5–104.2%) in serum, yet lack visual indication.^40^ GQDs-MWCNTs, relying on differential pulse voltammetry (DPV), reach an LOD of 0.87 nM and excel in real-sample recovery (98.7–105.8%), but their single electrochemical mode limits versatility.^41^ Other methods, such as FeS_2_-porous carbon (LOD: 0.015 μM) and CQDs-SPR (LOD: 0.1 pM), provide colorimetric or SPR-based detection, yet their linear ranges and matrix tolerance vary, with some showing moderate interference (*e.g.*, H_2_O_2_ for FeS_2_).^42,43^ In contrast, the PQD-COF Mats in this study integrate fluorescence (LOD: 0.3 fM) and EIS (LOD: 2.5 fM) with a visual green-to-pink shift *via* rhodamine B at DA >100 pM, achieving recoveries of 97.5–103.8% in serum and 97.9–99.7% in PC12 supernatant, suggesting a balanced performance across diverse metrics.

**Table 5:** Comparison of DA detection methods

Method	Mode(s)	LOD	Linear range	Specificity	Real sample recovery (%)	Visual detection	Reference
MIPs@CsPbBr_3_/CsPbI_3_	Fluorescence	1.9 nM	15–135 nM	High (Rhein-specific *via* MIPs)	Not tested (Rhein in herbs)	Yes (green-to-red shift)	39
N-GDQDs	Fluorescence + EIS	0.14 μM (F), 0.02 μM (E)	0.32–500 μM (F), 0.05–240 μM (E)	High (minimal interference from AA, UA)	96.5–104.2 (serum)	No	40
GQDs-MWCNTs	Electrochemical (DPV)	0.87 nM	0.005–100 μM	High (selective over AA, UA)	98.7–105.8 (serum, PC12 supernatant)	No	41
FeS_2_-porous carbon	Colorimetric + EIS	0.015 μM	0.05–1000 μM	Moderate (some interference from H_2_O_2_)	95.8–103.5 (serum)	Yes (TMB color change)	42
CQDs-SPR	SPR	0.1 pM	0.001–100 pM	High (selective over AA, glucose)	Not reported (serum, urine tested)	No	43
COF/Pt/MWCNT-COOH	Electrochemical (DPV)	0.23 nM	0.001–100 μM	High (selective over AA, UA)	96.8–102.5 (blood)	No	44
Au nanoelectrode arrays	Electrochemical (CV)	10 nM	0.05–100 μM	High (selective in neural cultures)	Not reported (PC12 supernatant tested)	No	45
Laccase-SPR	SPR	0.65 nM (0.1 ng mL^−1^)	0.065–1000 μM (0.01–189 μg mL^−1^)	High (enzyme-specific to DA)	Not reported (serum tested)	No	46
PQD-COF mats (this work)	Fluorescence + EIS with visual indication	0.3 fM (F), 2.5 fM (E)	1 fM to 10 pM (F), 10 fM to 500 μM (E)	High (<6% interference from AA, UA, *etc.*)	97.5–103.8 (serum), 97.9–99.7 (PC12)	Yes (green-to-pink with rhodamine B)	This study

Beyond sensitivity, the choice of detection platform often hinges on practical considerations like dynamic range and ease of use in real-world settings. Methods like COF/Pt/MWCNT-COOH (LOD: 0.23 nM) and Au nanoelectrode arrays (LOD: 10 nM) offer robust electrochemical detection with high specificity, yet their narrower linear ranges (*e.g.*, 0.001–100 μM and 0.05–100 μM, respectively) may limit applicability for tracking DA across physiological extremes.^44,45^ Laccase-SPR, with an LOD of 0.65 nM and a broad range (0.065–1000 μM), leverages enzyme specificity, but its reliance on SPR equipment reduces portability. The PQD-COF Mats, however, span an exceptionally wide linear range (1 fM to 10 pM for fluorescence, 10 fM to 500 μM for EIS), accommodating both trace-level detection and higher physiological concentrations.^46^ This adaptability, paired with minimal interference (<6% from AA, UA, *etc.*), positions the mats as a flexible tool, particularly when considering the added visual cue that simplifies preliminary assessments without sacrificing quantitative precision.

A key challenge in DA sensing is balancing analytical rigor with operational simplicity, especially for biological applications. While methods like CQDs-SPR and GQDs-MWCNTs demonstrate impressive LODs and selectivity, their lack of a visual component may necessitate additional instrumentation, potentially complicating field use. The incorporation of rhodamine B in the PQD-COF Mats not only enhances user-friendliness but also complements the dual-mode detection, a feature absent in most comparators. Furthermore, the mats' stability—retaining over 92% of fluorescence and R_ct_ after 30 days and 20 cycles—rivals or exceeds that of carbon-based systems, offering reliability for repeated measurements. While no single method universally outperforms others across all contexts, the combination of femtomolar sensitivity, broad dynamic range, and practical visual indication in this study provides a compelling framework that could inspire further refinements in neurotransmitter monitoring, subtly aligning with the evolving needs of both research and diagnostics.

## 4. Conclusions

This study introduces a pioneering dual-mode sensing platform utilizing CsPbBr_3_ PQD-integrated COF nanocomposites for ultrasensitive DA detection. By synergistically combining fluorescence quenching and EIS, the CsPbBr_3_-PQD-COF nanocomposites achieve remarkable limits of detection (0.3 fM for fluorescence, 2.5 fM for EIS) across an extensive linear range (1 fM to 500 μM). The platform's high sensitivity stems from the optoelectronic properties of CsPbBr_3_ PQDs and the π-conjugated COF scaffold, facilitating selective DA recognition through electron transfer and π–π stacking interactions. The incorporation of rhodamine B enhances practical utility by providing a visible green-to-pink shift at DA concentrations above 100 pM. Demonstrating excellent specificity (<6% interference from ascorbic acid, uric acid, *etc.*), stability (over 30 days), and real-sample performance (recovery rates of 97.5–103.8% in human serum and 97.9–99.7% in PC12 supernatant), this nanocomposite outperforms many existing methods in both sensitivity and versatility. The integration of femtosecond-level detection, a broad dynamic range, and visual indication positions the CsPbBr_3_-PQD-COF nanocomposites as a transformative tool for biosensing applications, with significant potential for real-time neurotransmitter monitoring and broader bioanalytical advancements in clinical and research settings.

## Data availability

All data supporting the findings of this study are available within the article and its ESI† files. Raw data, including high-resolution transmission electron microscopy (HR-TEM) images, X-ray diffraction (XRD) patterns, photoluminescence (PL) spectra, electrochemical impedance spectroscopy (EIS) measurements, and fluorescence quenching datasets, are presented in the figures and tables of the manuscript. Additional experimental details, such as synthesis protocols for covalent organic framework (COF) precursors and detailed characterization data, are provided in the ESI.† Any further data that support the plots and results reported in this work are available from the corresponding author (Hadi Noorizadeh, Department of Chemistry, Islamic Azad University, Tehran, Iran, Email: hadinoorizadehacademic@gmail.com) upon reasonable request.

## Conflicts of interest

We hereby declare that we have no conflict of interest regarding the content of this manuscript. All research and findings presented in this work are independent and have been conducted without any personal, financial, or professional bias.

## Supplementary Material

RA-015-D5RA02376A-s001
