# Supplementary material for: Ultrasensitive dopamine detection using CsPbBr3-PQD-COF nanocomposites: a synergistic fluorescence and EIS approach
Source: RSC Adv. 2025 Jun 4;15(24):18875–92. doi: 10.1039/d5ra02376a (PMC12136283; doi:10.1039/d5ra02376a)
Supplement: RA-015-D5RA02376A-s001 [file RA-015-D5RA02376A-s001.pdf]

Supplementary Information (SI) for RSC Advances.
This journal is © The Royal Society of Chemistry 2025

Synthesis Protocols for Covalent Organic Framework (COF) Precursors

This section provides detailed synthetic procedures and characterization data for 1,3,5-tris(4-aminophenyl)benzene (TAPB) and 2,5-dihydroxyterephthalaldehyde (DHTA), used as precursors for the covalent organic framework (COF) in the study titled "Ultrasensitive Dopamine Detection Using $CsPbBr_3$-PQD-COF Nanocomposites: A Synergistic Fluorescence and EIS Approach." The syntheses were performed following established protocols, and the products were characterized to confirm their structure and purity.

1. Synthesis of 1,3,5-Tris(4-aminophenyl)benzene (TAPB)

Materials

- 1,3,5-Tribromobenzene (98%, Sigma-Aldrich)
- 4-Aminophenylboronic acid pinacol ester (98%, Sigma-Aldrich)
- Tetrakis(triphenylphosphine)palladium(0) ($Pd(PPh_3)_4$, 99%, Sigma-Aldrich)
- Potassium carbonate (K_2CO_3, 99%, Merck)
- Toluene (anhydrous, 99.8%, Fisher Scientific)
- Ethanol (absolute, 99.9%, Fisher Scientific)
- Deionized water (resistivity 18.2 MΩ·cm, Milli-Q Advantage A10 system)
- Dichloromethane (DCM, 99.8%, Sigma-Aldrich)
- Magnesium sulfate ($MgSO_4$, anhydrous, 99%, Sigma-Aldrich)
- Nitrogen gas (99.999%, Air Liquide)

Procedure

TAPB was synthesized via a Suzuki-Miyaura cross-coupling reaction, adapted from reported methods [1]. In a 250 mL three-neck round-bottom flask equipped with a magnetic stirrer, condenser, and nitrogen inlet, 1,3,5-tribromobenzene (3.15 g, 10 mmol) and 4-aminophenylboronic acid pinacol ester (7.23 g, 33 mmol, 1.1 equiv per bromine) were dissolved in a degassed mixture of toluene (100 mL) and ethanol (50 mL). An aqueous solution of potassium carbonate (8.28 g, 60 mmol, in 50 mL deionized water) was added under continuous nitrogen purging. $Pd(PPh_3)_4$ (0.35 g, 0.3 mmol, 3 mol%) was introduced as the catalyst, and the mixture was degassed for an additional 15 minutes to remove residual oxygen. The reaction mixture was heated to 80 °C and stirred under reflux for 48 hours under a nitrogen atmosphere.
After cooling to room temperature, the organic layer was separated, and the aqueous layer was extracted with dichloromethane (3 × 50 mL). The combined organic extracts were dried over anhydrous $MgSO_4$, filtered, and concentrated under reduced pressure using a rotary evaporator. The crude product was purified by recrystallization from a toluene/ethanol mixture (3:1 v/v) to yield TAPB as a pale yellow solid (2.81 g, 80% yield).

Characterization

- **^{1}H NMR** (400 MHz, DMSO-d_6): δ 7.65 (s, 3H, central benzene), 7.38 (d, J = 8.4 Hz, 6H, Ar-H), 6.68 (d, J = 8.4 Hz, 6H, Ar-H), 5.21 (s, 6H, NH_2).

- **FTIR** (KBr, cm^{-1}): 3440, 3360 (N-H stretch, primary amine), 3030 (C-H aromatic), 1620 (C=C aromatic), 1510 (C-N).
- **Melting Point**: 320–322 °C (lit. 319–323 °C [1]).
- **Elemental Analysis**: Calculated for $C_{24}H_{21}N_3$: C, 81.57; H, 5.99; N, 11.89. Found: C, 81.50; H, 6.02; N, 11.85.

2. Synthesis of 2,5-Dihydroxyterephthalaldehyde (DHTA)
Materials

- 2,5-Dihydroxyterephthalic acid (98%, Sigma-Aldrich)
- Borane-tetrahydrofuran complex (BH_3·THF, 1 M in THF, Sigma-Aldrich)
- Phosphorus oxychloride ($POCl_3$, 99%, Sigma-Aldrich)
- N,N-Dimethylformamide (DMF, anhydrous, 99.8%, Sigma-Aldrich)
- Dichloromethane (DCM, 99.8%, Sigma-Aldrich)
- Sodium bicarbonate ($NaHCO_3$, 99%, Merck)
- Deionized water (resistivity 18.2 MΩ·cm, Milli-Q Advantage A10 system)
- Ethyl acetate (99.8%, Fisher Scientific)
- Magnesium sulfate ($MgSO_4$, anhydrous, 99%, Sigma-Aldrich)
- Nitrogen gas (99.999%, Air Liquide)

Procedure

DHTA was synthesized via a two-step process involving reduction of 2,5-dihydroxyterephthalic acid to the corresponding diol, followed by oxidation to the dialdehyde, adapted from established methods [2].

Step 1: Reduction to 2,5-Dihydroxy-1,4-benzenedimethanol

In a 500 mL three-neck round-bottom flask equipped with a magnetic stirrer and nitrogen inlet, 2,5-dihydroxyterephthalic acid (1.98 g, 10 mmol) was suspended in anhydrous THF (100 mL) under a nitrogen atmosphere. The mixture was cooled to 0 °C using an ice bath, and BH_3·THF (40 mL, 40 mmol, 1 M in THF) was added dropwise over 30 minutes with vigorous stirring. The reaction mixture was warmed to room temperature and stirred for 12 hours. The reaction was quenched by slow addition of methanol (20 mL) at 0 °C, followed by evaporation of the solvent under reduced pressure. The residue was dissolved in ethyl acetate (100 mL), washed with saturated $NaHCO_3$ (2 × 50 mL) and deionized water (50 mL), dried over $MgSO_4$, and concentrated to yield 2,5-dihydroxy-1,4-benzenedimethanol as a white solid (1.53 g, 90% yield).

Step 2: Oxidation to 2,5-Dihydroxyterephthalaldehyde

In a 250 mL three-neck round-bottom flask under nitrogen, 2,5-dihydroxy-1,4-benzenedimethanol (1.70 g, 10 mmol) was dissolved in anhydrous DMF (50 mL). The solution was cooled to 0 °C, and $POCl_3$ (4.6 mL, 50 mmol) was added dropwise over 20 minutes. The mixture was stirred at 0 °C for 1 hour, then heated to 60 °C and maintained for 6 hours. After cooling, the reaction was quenched by pouring into ice-cold water (200 mL) and neutralized with

saturated $NaHCO_3$ until pH ~7. The precipitate was filtered, washed with deionized water (3 × 50 mL), and recrystallized from ethanol to yield DHTA as a bright yellow solid (1.33 g, 80% yield).

Characterization

- **^{1}H NMR** (400 MHz, DMSO-d_6): δ 10.25 (s, 2H, CHO), 9.85 (s, 2H, OH), 7.35 (s, 2H, Ar-H).
- **FTIR** (KBr, cm^{-1}): 3300 (O-H stretch), 2870 (C-H aldehyde), 1680 (C=O aldehyde), 1510 (C=C aromatic), 1230 (C-O).
- **Melting Point**: 192–194 °C (lit. 190–195 °C [2]).
- **Elemental Analysis**: Calculated for $C_8H_6O_4$: C, 57.84; H, 3.64; O, 38.52. Found: C, 57.80; H, 3.67; O, 38.49.

References

[1] Yaghi OM, Kalmutzki MJ, Diercks CS. *Introduction to Reticular Chemistry: Metal-Organic Frameworks and Covalent Organic Frameworks*. Wiley-VCH, 2019.
[2] Wang J, Dai Z, Wen H, Tan H, Shi Y, Huang L, Yu W, Liang S, Yang J, Yuan S. Hydroxyl-Functionalized Ultrathin Covalent Organic Framework Membrane for Fast H_2/CO_2 Separation. *ACS Sustainable Chemistry & Engineering*. 2025 May 14.
